# To Save the Edge of Sterilized Instruments

**Published:** 1891-01

**Authors:** John S. Miller

**Affiliations:** Philadelphia


### To Save the Edge of Sterilized Instruments.

To the Editor.

*Sir:* While attending Prof. von Bergmann's surgical clinic at Berlin recently, the following demonstration was made, which will certainly interest your surgical readers.

To render instruments perfectly aseptic, and to preserve the cutting edges from oxidation, they are boiled for five minutes in a one per cent. solution of carbonate of soda. They can remain in this solution indefinitely without rusting or dulling the cutting edge. When required for operation they are taken out, dried with a sterilized piece of gauze, and handed to the operator. Whenever, in course of the operation, they come in contact with any thing not aseptic, all that is required to re-sterilize them, is to dip them for a few seconds into the boiling solution of sodium bicarbonate. Yours truly,

Philadelphia. John S. Miller, M.D.

